# Isolation, identification, and serotyping of *Avibacterium paragallinarum* from quails in Indonesia with typical infectious coryza disease symptoms

**DOI:** 10.14202/vetworld.2018.519-524

**Published:** 2018-04-23

**Authors:** Agnesia Endang Tri Hastuti Wahyuni, Charles Rangga Tabbu, Sidna Artanto, Dwi Cahyo Budi Setiawan, Sadung Itha Rajaguguk

**Affiliations:** 1 Department of Microbiology, Faculty of Veterinary Medicine, University of Gadjah Mada, Yogyakarta, Indonesia; 2 Department of Pathology, Faculty of Veterinary Medicine, University of Gadjah Mada, Yogyakarta, Indonesia; 3 Department of Pharmacology, Faculty of Veterinary Medicine, University of Gadjah Mada, Yogyakarta, Indonesia; 4 Department of Microbiology, Faculty of Veterinary Medicine, University of Gadjah Mada, Yogyakarta, Indonesia

**Keywords:** antibiotic sensitivity test, *Avibacterium paragallinarum*,infectious coryza, nicotinamide adenine dinucleotide-independent

## Abstract

**Background and Aim:**

Infectious coryza (IC) or snot is an infectious upper respiratory disease affecting chickens and birds, including quails, and it is caused by *Avibacterium paragallinarum*. The symptoms of IC are facial swelling, malodorous nasal discharge, and lacrimation. This study aimed to isolate, identify, and serotype the *A. paragallinarum* of snot in quails and to determine the sensitivity and resistance to several antibiotics.

**Materials and Methods:**

Nine quails from Yogyakarta, Indonesia with typical snot disease symptoms were used in this study. The nasal swab was obtained and directly streaked onto a chocolate agar plate and blood agar plate (BAP), then incubated in 5% CO_2_ at 37°C for 24-48 h. *Staphylococcus* spp. was cross-streaked onto the BAP to show the satellite growth. The observation of the morphology of the suspected colony, Gram staining, and biochemical tests (catalase test, oxidase test, urease test, peptone test, and carbohydrate fermentation such as maltose, mannitol, lactose, and sorbitol) are done to identify the species of bacteria. This research also detects the serovar of *A. paragallinarum* using hemagglutination inhibition test.The antibiotic sensitivity tests were also performed using several antibiotics against five *A. paragallinarum* isolates that were cultured on Mueller–Hinton Agar and added with antibiotic discs, then incubated in 5% CO_2_ at 37°C for 24–48 h.

**Results:**

Five isolates out of nine suspected isolates (55.5%) were *A. paragallinarum*. The growth of isolates from quails did not depend on the nicotinamide adenine dinucleotide (NAD) (NAD-independent). Sensitivity test was done using the five identified *A. paragallinarum* isolates, results showed that they were 100% sensitive to amoxicillin (AMC) and ampicillin (AMP); 100% resistant toward amikacin (AK), erythromycin (E), gentamycin (CN), and tetracycline (TE); 80% resistant toward kanamycin (K) and trimethoprim (W); 60% resistant toward chloramphenicol (C); and 20% toward enrofloxacin (ENR). The antibiotics that have an intermediate sensitivity (in between sensitive and resistant) were ENR and K, 80% and 20%, respectively. Three out of five *A. paragallinarum* isolates were identified as serovar B of *A. paragallinarum* using HI test.

**Conclusion:**

Five out of nine isolates (55.5%) from quails with typical IC disease symptoms identified as *A. paragallinarum* and sensitive toward AMC and AMP. Three out of five *A. paragallinarum* isolates were identified as serovar B.

## Introduction

Infectious coryza (IC) or snot is an infectious upper respiratory disease affecting poultry [1-3]. This disease is caused by *Avibacterium paragallinarum*, which was previously known as *Haemophilus paragallinarum* [4]. Some clinical signs that are commonly seen in IC are rhinitis, facial swelling or edema, anorexia, and retarded growth in young poultry [5-7]. The disease is very important in the chicken farm industry in developed and developing countries, including Indonesia [8]. The large economic losses due to IC such as increased number of culling, decreased egg production (10-80%), decreased body weight, stunting growth, and some mortality (2-10%) [4]. This disease can be found worldwide, especially in tropical countries. The diagnosis can be based on a history of rapid disease spread, clinical symptoms, and pathological changes caused by snot. Whereas, the definitive diagnosis of this disease can be made by isolating and identifying the causative bacteria in the acute stage of the disease and isolated directly using nasal swab from infraorbital sinus [9-11]. Advance molecular techniques such as HPG2-polymerase chain reaction (PCR), enterobacterial repetitive intergenic consensus-PCR, and 16 s rRNA sequencing can also be used for rapid and accurate identification of bacterium [12-14]. There are three major recognized *A. paragallinarum* serovars, which are serovar A, B, and C. Limited reports regarding the serovars of *A. paragallinarum* in Indonesia are available. This research was done to detect the serovar of *A. paragallinarum* in quail using hemagglutination inhibition (HI) test. It has been found that all three serovars of *A. paragallinarum* show variation in the virulence [15].

Snot commonly reported in the layers and broilers, compared to the quails [16]. The quails are susceptible to *A. paragallinarum* infection, but the isolation and characterization of this bacterium were rarely performed. In Indonesia, the snot reports in quails have been increasing in the past few years [17]. Many antibiotics have been used to treat snot, but many of them could only lower the severity of the disease without actually curing the disease completely. If the treatment was repeated, it would lead to resistance to certain antibiotics [11]. Therefore, the sensitivity tests of *A. paragallinarum* toward antibiotics are important to determine appropriate treatment regime for this disease.

This study aimed to isolate and identify *A. paragallinarum* in quails that showed typical symptoms of snot and to determine the sensitivity and resistance toward several antibiotics.

## Materials and Methods

### Ethical approval

No ethical clearance was needed for this research since the samples were obtained as per standard sample collections procedure without any harm to animals. Nasal swab samples were obtained from live animals without anesthetic treatment.

### Sample collection

The samples were collected from nine quails with typical facial edema and discharges from nasal and infraorbital sinuses. The quails were from quail farm in Yogyakarta, Indonesia. The study was done in Microbiology Laboratory, Faculty of Veterinary Medicine, University of Gadjah Mada.

### Isolates and culture conditions

There were nine quails showing typical clinical signs of IC. The samples collected were from nasal sinus discharge and infraorbital sinuses. The initial inoculation was performed onto chocolate agar plate (CAP) because this medium supplies V factor which is necessary for *A*. *paragallinarum* growth and then incubated in the 5% CO_2_ incubator at 37°C for 24-48 h. CAP medium can produce more *A. paragallinarum* colonies [18].

The suspected colony of *A. paragallinarum* was then cultured onto the blood agar plate (BAP), added with feeder bacteria, *Staphylococcus* sp. This was done by streaking inoculation loop perpendicular to the *A. paragallinarum* streaks (cross-streak) [19]. Afterward, the plates were incubated in the 5% CO_2_ incubator at 37°C for 24-48 h[20]. The colony with satellite growth was then stained using Gram staining method, tested for catalase test, oxidase test, motility test, urease test, and carbohydrate fermentation test [5,21,22].

### Serotyping

Three isolates were serotyped according to the Kume’s scheme. The Kume serotyping was performed using antisera [23]. Antisera for HI test were provided by SHS International^®^, which are serotype A (221 strain) and serotype B (Spross strain) from Aburahi Lab, Japan; while serotype C (Modesto strain) is from Animal Research Institute, Australia. Serotype C (Modesto strain) is from Animal Research Institute, Australia.

### Preparation of antigens for serotyping tests

*A. paragallinarum* strains were microaerophilically grown in brain–heart infusion broth, supplemented with nicotinamide adenine dinucleotide (NAD) as supporting growth factor. After incubation, the broth was centrifuged twice at 3000× *g* for 15 min at 4°C. The supernatant is removed, and phosphate-buffered saline (PBS) is homogenized with sediment before it is sonicated for 3 min. The sonicated sediment is then added with the adequate amount of NaCl 0.15 M so that the cloudiness of the solution matches with the turbidity of 0.5 McFarland standard solutions [24]. The pellet was then resuspended in 4 ml of PBS with thimerosal (100 mg/ml) and kept at 4°C until usage.

### Preparation of chicken erythrocytes

Glutaraldehyde-fixed erythrocyte (GA-fixed RBC) suspension (30%) in distilled water added with 100 mg/ml of thimerosal was prepared and kept in the dark at 4°C. When needed, this solution was diluted 1:30 in a working buffer consist of PBS (pH 7.2) with 0.1% of bovine serum albumin and 0.001% of gelatin.

### Hemagglutination (HA) tests

The HA titers of the antigens were determined with 40 µl of reagent in a microdilution method. Doubling dilutions of antigen were made with working buffer. GA-fixed RBC (1% in working buffer) was added to each well, and the plates were incubated for 1 h at room temperature until the RBC control forms a pinpoint shape. The concentration of antigens is adjusted as a result of 4 HA unit is achieved.

### HI test

Forty-microliter (40 µl) of doubling dilutions of antisera at 1:20-1:1.280 were prepared in working buffer. An equal amount of 4 HA unit antigen followed by 40 µl of 1% GA-fixed RBC was added to each well. Plates were read after 1 h at room temperature. The HI titer was the most diluted antiserum that inhibits HA. The hemagglutinin serovar of each isolate was assigned according to the highest HI titer.

### Antimicrobial sensitivity test

The antimicrobial sensitivity test was performed using Chukiatsiri *et al*. [25] method with some modifications. Isolates that have been identified as *A. paragallinarum* were tested against most commonly used antibiotic disc. *A. paragallinarum* cultured into the TM/SN broth and incubated in the 5% CO_2_ incubator at 37°C for 24-48 h. The bacteria suspension was made equivalent to 0.5 McFarland turbidity standard and then spread onto Mueller–Hilton Agar (MHA oxoid™) medium with sterile swab. Antibiotic disc amoxicillin (AMC), ampicillin (AMP), chloramphenicol (C), trimethoprim (W), amikacin (AK), erythromycin (E), gentamycin (CN), tetracycline (TE), kanamycin (K), and enrofloxacin (ENR) were placed on the agar surface. The medium was incubated in the 5% CO_2_ incubator at 37°C. The zone of inhibition was observed after 24-48 h and the diameter of inhibition zone was measured and recorded in millimeters. The susceptibility category (sensitive, intermediate, or resistant) was determined by comparing the zone of antibiotic with the zone diameter breakpoint, as recommended by CLSI [26].

## Results

**Figure-1:**
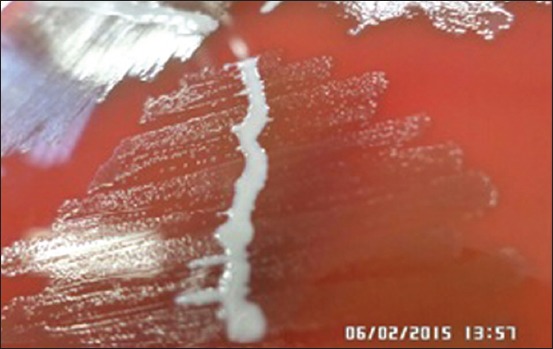
Colony morphology of *Avibacterium paragallinarum* (A) in BAP added with *Staphylococcus* spp (B) culture.

The observation of the nine quails that were suspected IC disease showed that all of them had facial edema and produced malodorous mucopurulent nasal discharge. From the samples, five of them (55.5%) were *A. paragallinarum*. The data of the quails that showed typical clinical signs of snot and their colony morphology can be seen in Table-1. The addition of *Staphylococcus* spp. onto BAP medium showed that *A. paragallinarum* isolates from quails were NAD-independent (Figure-1). Five isolates (Q3, Q4, Q5, Q7, and Q9) showed biochemical characteristics of *A. paragallinarum*,i.e., negative catalase, negative oxidase, negative urease, negative in indole test, and capable to fermenting carbohydrate (Table-2).

**Table-1:** Quail’s data showing typical snot clinical signs with their colony morphology on CAP.

Sample code	Clinical signs		Suspected colony morphology		
	
	Facial edema	Malodorous nasal discharge	Color		Shape
			
			Smooth, transparent	White, compact	Circular
Q1	+	+	+	+	+
Q2	+	+	+	+	+
Q3	+	+	+	+	+
Q4	+	+	+	+	+
Q5	+	+	+	+	+
Q6	+	+	+	+	+
Q7	+	+	+	+	+
Q8	+	+	+	+	+
Q9	+	+	+	+	+

CAP=Chocolate agar plate

**Table-2:** Biochemical test results of suspected colonies isolated from quails showing snot symptoms.

Sample code	Shape	K	O	U	I	Mt	M	Ma	L	S
Q1	Coccobacillus	+	+	+	+	−	+	+	−	+
Q2	Coccobacillus	+	−	+	+	+	−	−	−	−
Q3+	Coccobacillus	−	−	−	−	−	+	+	+*	+
Q4+	Coccobacillus	−	−	−	−	−	+	+	+	+
Q5+	Coccobacillus	−	−	−	−	−	+	+	+	+
Q6	Coccobacillus	+	−	−	−	+	−	−	+	−
Q7+	Coccobacillus	−	−	−	−	−	+	+	+	+
Q8	Coccobacillus	+	−	−	−	+	−	−	+	−
Q9+	Coccobacillus	−	−	−	−	−	+	+	+*	+

K=Catalase, I=Indole, Ma=Maltose, O=Oxidase, Mt=Motility, L=Lactose, U=Urease, M=Mannitol, S=Sorbitol, ^+^A. paragallinarum,

* Low fermentation, A. paragallinarum=Avibacterium paragallinarum

The 4 HA unit antigen was used to perform HI test immediately as further prolongation may cause an alteration in the concentration of antigen. Due to antisera limitation, HI test is only done on three isolates, i.e., Q5, Q7, and Q9 isolate. The result showed that the three isolates were serovar B strain.

The sensitivity test of *A. paragallinarum* shows different result toward all the antibiotics (Table-3). The sensitivity level toward AMC and AMP was 100%, toward C and W was 20%, whereas these bacteria were 100% resistant toward AK, E, CN, and TE; 80% toward K and W; 60% toward C; and 20% toward ENR. The antibiotics that have an intermediate sensitivity (in between sensitive and resistant) were ENR and K, 80% and 20%, respectively.

**Table-3:** Result of sensitivity test of A. paragallinarum isolated from quails toward 10 antibiotics.

Isolate code	Inhibition zone (mm)										

	AK	AMC	ENR	AMP	C	E	CN	K	TE	W
Q3	0 (R)	32 (S)	22 (I)	30 (S)	10 (R)	0 (R)	9 (R)	0 (R)	0 (R)	0 (R)
Q4	10 (R)	31 (S)	11 (R)	27 (S)	24 (S)	10 (R)	0 (R)	0 (R)	0 (R)	0 (R)
Q5	10 (R)	30 (S)	18 (I)	28 (S)	10 (R)	10 (R)	10 (R)	0 (R)	0 (R)	10 (R)
Q7	10 (R)	18 (S)	22 (I)	27 (S)	16 (I)	10 (R)	11 (R)	15 (I)	9 (R)	28 (S)
Q9	10 (R)	30 (S)	22 (I)	24 (S)	12 (R)	10 (R)	10 (R)	0 (R)	0 (R)	10 (R)

R=Resistant, I=Intermediate, S=Sensitive, A. paragallinarum=Avibacterium paragallinarum, AK=Amikacin, AMC=Amoxicillin, ENR=Enrofloxacin, AMP=Ampicillin, C=Chloramphenicol, E=Erythromycin, CN=Gentamycin, K=kanamycin, TE=Tetracycline, W=Trimethoprim

## Discussion

The clinical signs showed in the chicken suspected IC are facial swelling or facial edema, discharge from nasal sinuses, conjunctivitis, and decreased egg production [27-29]. Whereas infected quails that show typical signs would also produce foul smell in chronic infection [11]. The quails are susceptible to this disease in all ages [22].

**Figure-2:**
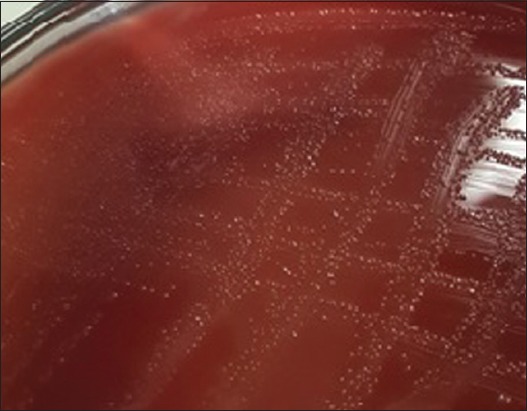
Morphology colony of *Avibacterium paragallinarum* in CAP

There were two colony morphologies on CAP from the first culture of samples (Table-1). The *A. paragallinarum* colony on CAP is circular, transparent, and smooth dewdrops (Figure-2).. This bacterium had relatively slow growth and could be detected after being incubated for 36-48 h [13]. Besides *A. paragallinarum*, *Staphylococcus* sp. can also grow on this medium and could be well observed with white circular morphology. Gram stain of *A. paragallinarum* showed that bacteria were coccobacilli morphology and red color (Gram-negative). The result obtained matches with the study reported by Akhter *et al*. [18] and Priya *et al*. [30]. Colonies with morphological characteristics leading to *A. paragallinarum* colonies were recultured until got pure colony.

The suspected colony was isolated to BAP medium and added with *Staphyococcus* spp. as bacterial feeder [31,32]. The *A. paragallinarum* that needs V factor would grow alongside the bacteria feeder and form satellite colony [3,4,33,34], while some *A. paragallinarum* species could grow even without V factor. The addition of *Staphylococcus* sp. onto BAP medium in this study showed that *A. paragallinarum* isolates from quails were NAD-independent. Thecolony had bigger size (1-2 mm) compared to NAD-dependent *A. paragallinarum* isolates (0.3 mm) and did not show any satellite colony [25]. The NAD-independentisolates had lower virulence level compared to NAD-dependent isolates, but the differences have not yet been explained [31].

Five isolates (Q3, Q4, Q5, Q7, and Q9) showed biochemical characteristics of *A. paragallinarum*, i.e., negative catalase, negative oxidase, negative urease, negative in indole test, and capable to fermenting all carbohydrate that used, which are also reported by Blackall and Soriano [19]. Two of five (Q3 and Q9) isolates showed low fermentation of lactose. The same results were reported by Akhter *et al*. [5], that ability of fermenting lactose is variable. Four other identified isolates showed differences in the ability of fermenting carbohydrates. The other biochemical tests also do not confirm that the four isolates are *A. paragallinarum*.

Hemagglutinin (HA) proteins have an important role in the pathogenesis and immunopathogenesis of *A. paragallinarum* [35]. The HA test is necessary before HI test as 4 HA unit of antigen concentration is required for HI test and also to confirm the ability of bacteria to agglutinate erythrocytes [23]. The 4 HA unit antigen was used to perform HI test immediately as further prolongation may cause an alteration in the concentration of antigen. HI test is normally done to detect the minimum antibody titer needed to inhibit the action of HA. According to Blackall and Soriano [19], two separate serotyping schemes were used for detection of *A. paragallinarum* on the serological level, Page scheme (1962) and Kume scheme (1983). The HA-HI test is chosen based on the availability of antisera. Due to antisera limitations, HI test was only done on three isolates, i.e., Q5, Q7, and Q9. The result showed that three isolates were serovar B.

Sensitivity of *A. paragallinarum* to AMC and AMP was 100%, to C and W was 20%, whereas resistance of *A. paragallinarum* to AK, E, CN, and TE was 100%; K and W was 80%; C was 60%; and ENR was 20%. Antibiotics that had intermediate sensitivity were ENR and K, 80% and 20% respectively. The resistance level toward erythromycin was more than 75% similar to other studies [25,36], whereas the high level of resistance toward tetracycline also reported by Thenmozi and Malmarungan [22] that occurred up to 70%. According to Rajurkar *et al*. [37], six *A. paragallinarum* in India used in their studies were all resistant to tetracycline. From anamnesis obtained, it was clear that the quails already obtained antibiotic treatment of enrofloxacin and neomycin. The resistance level toward tetracycline occurred in Indonesia because tetracycline is commonly used in the field to combat the snot disease, besides the erythromycin and sulfonamide [38].

## Conclusion

Five out of nine isolates (55.5%) from quails with typical IC disease symptoms identified as *A. paragallinarum* and sensitive to AMC and AMP. Three out of five *A. paragallinarum* isolates were identified as serovar B. Isolation, identification, and serotyping of *A. paragallinarum* in this study are important to provide solutions to the control coryza disease. From reports of field cases in Indonesia, commercial vaccine has not been able to cover the incidence of IC. Serotyping of *A. paragallinarum* is important to identify the serovars that commonly cause coryza infection in quail, therefore, serovar-specific vaccination to prevent occurrence of coryza in quail can be carried out more effectively. While antibiotic sensitivity test provides useful suggestion for appropriate treatment that is effective and efficient against the bacterial infection. Molecular characterization and serotyping of *A. paragallinarum* with multiplex PCR will be researched on the next project.

## Authors’ Contributions

AETHW contributed in isolation and identification process of *A. paragallinarum*,serotyping analysis. CRT contributed in the detection of clinical symptoms of snot in the farm. SA contributed in the serotyping test. DCBS contributed in antibiotic sensitivity test. SIR contributed in collecting samples. All authors read and approved the final manuscript.
